# Snails in the sun: Strategies of terrestrial gastropods to cope with hot and dry conditions

**DOI:** 10.1002/ece3.5607

**Published:** 2019-09-30

**Authors:** Mona Schweizer, Rita Triebskorn, Heinz‐R. Köhler

**Affiliations:** ^1^ Animal Physiological Ecology Institute of Evolution and Ecology University of Tübingen Tübingen Germany; ^2^ Steinbeis Transfer Center for Ecotoxicology and Ecophysiology Rottenburg Germany

**Keywords:** estivation, heat stress, Hsp, land snail, oxidative stress, polymorphism, shell coloration, stress ecology

## Abstract

Terrestrial gastropods do not only inhabit humid and cool environments but also habitat in which hot and dry conditions prevail. Snail species that are able to cope with such climatic conditions are thus expected to having developed multifaceted strategies and mechanisms to ensure their survival and reproduction under heat and desiccation stress. This review paper aims to provide an integrative overview of the numerous adaptation strategies terrestrial snails have evolved to persist in hot and dry environments as well as their mutual interconnections and feedbacks, but also to outline research gaps and questions that remained unanswered. We extracted relevant information from more than 140 publications in order to show how biochemical, cellular, physiological, morphological, ecological, thermodynamic, and evolutionary parameters contribute to provide an overall picture of this classical example in stress ecology. These mechanisms range from behavioral and metabolic adaptations, including estivation, to the induction of chaperones and antioxidant enzymes, mucocyte and digestive gland cell responses and the modification and frequency of morphological features, particularly shell pigmentation. In this context, thermodynamic constraints call for processes of complex adaptation at varying levels of biological organization that are mutually interwoven. We were able to assemble extensive, mostly narrowly focused information from the literature into a web of network parameters, showing that future work on this subject requires multicausal thinking to account for the complexity of relationships involved in snails' adaptation to insolation, heat, and drought.

## Graphical abstract

Mechanisms of terrestrial snails to cope with radiation, heat, and drought range from behavioral and metabolic adaptations, including estivation, to the induction of chaperones and antioxidant enzymes, mucocyte and digestive gland cell responses and the modification and frequency of morphological features, particularly shell pigmentation. In this context, thermodynamic constraints call for processes of complex adaptation at varying levels of biological organization which are mutually interwoven. Future work on this subject therefore requires multicausal thinking to account for the high complexity of relationships.

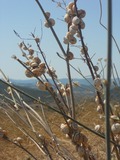

## 1 INTRODUCTION

Along with habitat loss, environmental disasters and freezing conditions, high temperature, and drought are among the major threats faced by terrestrial gastropods (Nicolai & Ansart, 2017). Many people might think of a typical land snail habitat as humid, cool, and shaded. Nevertheless, a number of pulmonate snail species live in arid, semiarid, and Mediterranean regions, where the climate is, at least temporarily, dominated by high ambient temperatures and low humidity (Mizrahi, Heller, Goldenberg, & Arad, 2010). Rising temperature and concomitant drought, however, rapidly bring ectotherms close to critical limits, particularly species that are not able to rapidly alter their distribution, such as land snails (Dillon, Wang, & Huey, 2010). In arid climate, there are times in which more than a year may pass between rains (Schmidt‐Nielsen, Taylor, & Shkolnik, 1971). The most severe problems caused by such hostile climates are thermal deaths as a result of high temperature and consequent desiccation (McQuaid, Branch, & Frost, 1979; Schmidt‐Nielsen et al., 1971), which may, occasionally, cause periodic mass mortalities even in regions of moderate climate (Nicolai, Filser, Lenz, Bertrand, & Charrier, 2011). Nevertheless, several species occur in arid and semiarid habitats at large population densities, for example, *Sphincterochila boissieri*, *Sphincterochila zonata*, *Sphincterochila cariosa*, *Theba pisana*, *Cernuella virgata*, *Xeropicta derbentina*, and *Trochoidea seetzeni*, among others (Dittbrenner et al., 2009; Mizrahi et al., 2010; Schmidt‐Nielsen et al., 1971; Yom‐Tov, 1971). In populations that are abundant in arid or semiarid regions, heat‐associated mortality is usually rather low (reviewed by Nicolai & Ansart, 2017). *Sphincterochila* species may even be found completely exposed to sun and heat, estivating on desert surfaces which, in mid‐summer, can reach up to 70°C. Depending on the extent of its exposure, *Sphincterochila boissieri*, which occurs in deserts of Israel and Egypt, survives internal temperatures between 50 and 55°C. Furthermore, this desert snail is capable of reducing its water loss to a minimum during estivation, so it can endure prolonged periods without rain and any other external water supply (Schmidt‐Nielsen et al., 1971; Yom‐Tov, 1971). Tolerance to such extreme conditions, however, seems to be an exception, as other arid or semiarid species like *Cernuella virgata*, *Cochlicella acuta*, or *Theba pisana* do not survive at 55°C air temperature for more than a few hours, which has led to trials to control snails in South Australian crops by knocking them down on the hot soil surface in summer—however, with varying success (Kempster & Charwat, 2003). The upper lethal limit of Mediterranean Theba pisana is 50°C (Cowie, 1985).

Obviously, extremely effective adaptations have evolved that ensure survival of land snails even in very dry and hot environments. Many studies have been conducted casting spotlights on various mechanisms and strategies that enable land snails to cope with such conditions. In this context, thermodynamic aspects are intermingled with physiological, morphological, cellular, and molecular traits which, on the one hand, are variable within the reaction norms but also are subject to selection. In some cases, traits of various kinds seem to influence one another reciprocally in a recursive procedure, reflecting the complexity of the entire picture, which we have only partly understood so far. Thus, this paper not only aims at summarizing existing knowledge on the strategies evolved but also combines information from various fields to provide an integrative and holistic view of the interactions that are associated with heat‐ and drought‐tolerating land snails. We also outline ambiguities and knowledge gaps that should be addressed in future research. Our work exclusively focuses on shelled terrestrial pulmonates, as slugs are far more susceptible to heat and drought and not of particular relevance in arid and semiarid environments. We also exclude aquatic snails such as Patelloidea and Littorinoidea, which, as intertidal snails, endure extremely variable environmental conditions that are quite different from those experienced by terrestrial species.

## 2 BEHAVIORAL AND PHYSIOLOGICAL ADAPTATIONS

The behavior and metabolism of terrestrial snails are closely connected and instantaneously depend on the thermodynamics of radiation (insolation), conduction (energy transfer by contact to surfaces), convection (atmospheric flow), and evaporative cooling (resulting in loss of water). On the one hand, the active choice of an optimal position of the body which should minimize its heating up during sunny days requires unimpeded metabolic activity which, in turn, further increases the body temperature. On the other hand, heat‐induced estivation enables survival by downregulation of the entire metabolism but prevents active movement and, thus, behavioral responses until the snail's arousal.

### 2.1 Burying and climbing

**Figure 1:**
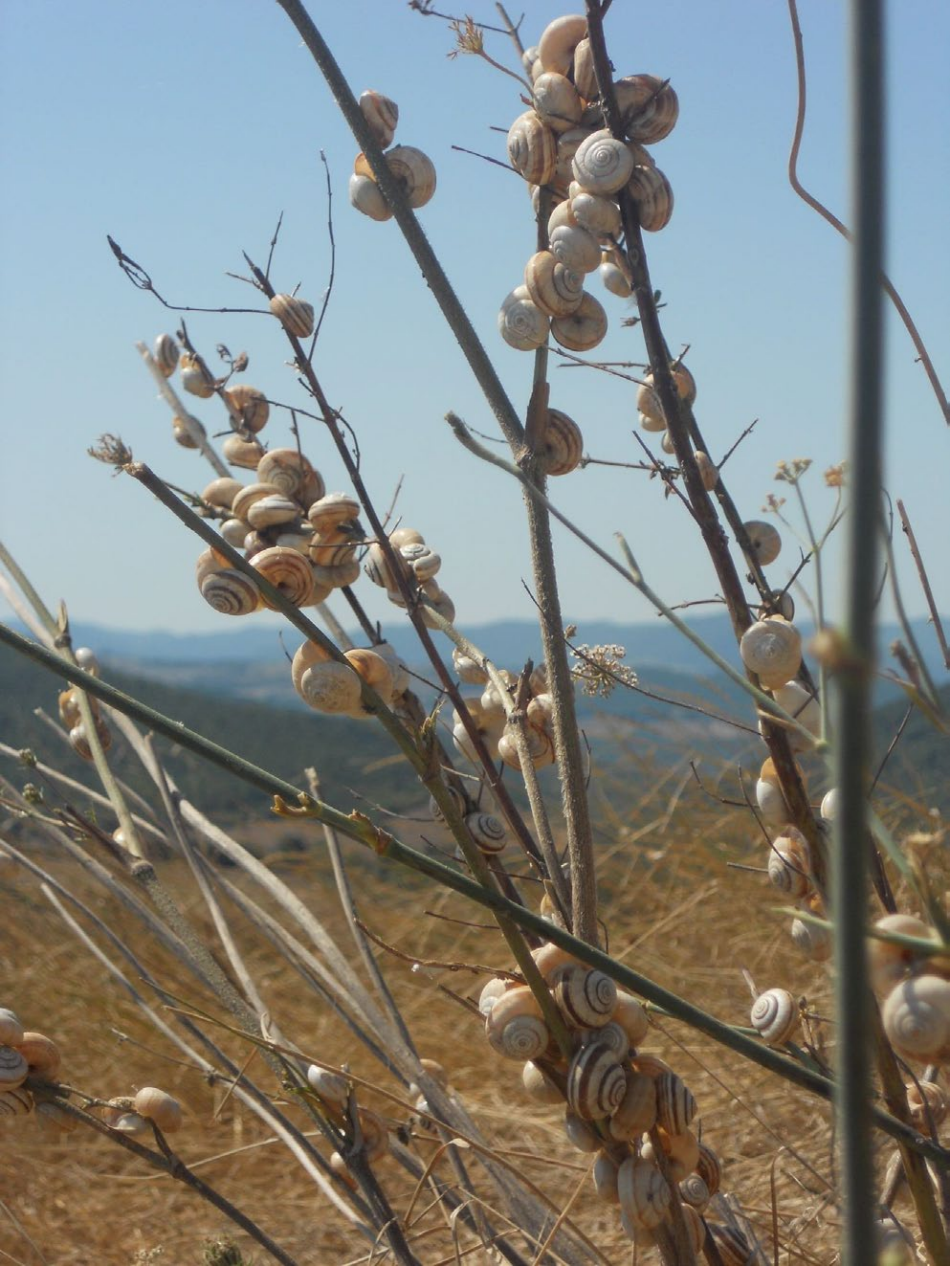
Different shell color morphs of *Cernuella virgata* (Hygromiidae) escaping from extreme soil surface temperatures by climbing a shrub, about 3 km south of Volterra, Tuscany, Italy (photograph by H‐R.K.)

Several behavioral strategies that contribute to avoiding hyperthermia and retaining body water have evolved in terrestrial snails. The simplest strategy is to stay in the shade, if shade‐giving vegetation, etc. exists. *Oxyloma retusa* actively moves to shaded regions to prevent its body temperature from reaching lethal levels (Riddle, 1990). *Cepaea vindobonensis* chooses resting sites that are well protected from direct solar radiation (Staikou, 1999). Another way to avoid solar radiation and the associated high temperatures is to restrict activity to favorable daytime periods, which are usually the night, early mornings, and/or later in the evening. This has been explicitly reported, for example, for *Theba pisana* (Cowie, 1990) and *Cepaea vindobonensis* (Staikou, 1999), but is commonly known for terrestrial gastropods. Consequently, snail species that live in arid or semiarid environments need to cope with situations during daytime when solar irradiation is strong and temperature is high, particularly at the soil surface. In the absence of vegetation, a behavioral strategy, especially for estivation, is to bury below the sun‐exposed surface. The desert snail *Sphincterochila boissieri* buries itself in the ground to a depth of 1–5 cm in summer, thereby protecting itself from intense solar radiation (Yom‐Tov, 1971). *Cepaea vindobonensis* estivates in the soil during the hot months of the year (Staikou, 1999). Other snail species like *Theba pisana*,* Xeropicta derbentina*, or *Cernuella virgate*, instead of burying, move upwards (Figure 1). By climbing tall objects, for example, grass blades, plant stalks, trees, fences, or artificial poles extreme temperatures at the soil surface can be avoided (Cowie, 1985; McQuaid et al., 1979). Although, at first glance, climbing sun‐exposed objects seems to be rather disadvantageous for the snails, as such behavior results in an even stronger exposure to solar radiation, McQuaid et al. (1979) showed the air temperature in sunny, open habitats to decrease with increasing distance from the ground. In the middle of a hot day in South Africa, the authors measured a difference in temperature of 8°C between 0 and 100 cm above the soil surface. In vegetation, conditions are similar but not as dramatic (Cowie, 1985). McQuaid et al. (1979) compared sheltered and exposed sites with the result that air temperatures at 0 cm were lower at sheltered than at exposed sites, but conditions were reversed at 50 cm where temperatures at sheltered sites were higher. A reason for this phenomenon could lie in thermal convection that may influence these habitats in different ways (Seuront, Ng, & Lathlean, 2018) but this physical principle never has been investigated thoroughly with respect to the thermodynamics of snails. Nevertheless, it is obvious that in radiation‐exposed sites like grassland, snails usually estivate some distance above the ground, often close to the tips of the grass blades. Such behavior has often been reported and has been well documented in studies of, for example, McQuaid et al. (1979) and Di Lellis et al. (2012). Snails in sheltered habitats do not seem to climb up that high even in the presence of bushes that are much larger than grasses (Cowie, 1985). Additional factors contribute to the determination of the optimal height for maintaining the snails' body temperature within critical limits including air temperature and insolation, as well as soil surface temperature and convective cooling, and the influence these factors have on the individual snail may be influenced by body orientation and microhabitat selection (Cowie, 1985; McQuaid et al., 1979). Using multiple regression modeling, Di Lellis et al. (2012) found the height above the ground and some orientation of the body to be apparently advantageous for particular morphs of *Xeropicta derbentina* under particular conditions, but it still remains unclear whether positive aspects of a distinct orientation of the body/shell to the sun can be generalized, and whether there are an optimal position and height above the ground at all. One may think of a trade‐off between increasing thermal benefits and increasing metabolic costs in this context but this has not been addressed in detail. Jones (1982) speculated that differences in the individual behavior of different morphs may contribute to the maintenance of polymorphisms (with respect to shell pigmentation) in *Cepaea nemoralis*. Also, different populations of the same species can have different thermal tolerances and optima, as shown for European *Theba pisana* populations along a longitudinal gradient (Cowie, 1985) and Chilean *Cornu aspersum* populations which exhibited increasing thermal optima with decreasing latitudes of the sites they originated from (Gaitán‐Espitia, Belen Arias, Lardies, & Nespolo, 2013).

### 2.2 Clustering

A typical example of the influence of ecological factors on snail behavior is clustering, which, as described by McQuaid et al. (1979) for *T. pisana*, results in a reduction of the body temperatures of the inner individuals of a cluster and, in turn, also in a decrease of the substrate temperature beneath. To achieve at least some cooling effect even in very exposed spots, snails often cluster in large numbers. Preference for the more shaded sides of substrates for estivation (Cowie, 1985) was also shown to help in adjusting insulation. Furthermore, McQuaid et al. (1979) observed that most *T. pisana* individuals estivated with the shell mouth directed upwards which results in the last whorl's air space being placed between the insulation barrier (the shell) and the snail's body. They concluded that this behavior helps in reducing heat uptake by conduction from the substrate as the heat flow can be dissipated via the air. It is, however, unclear whether the strategy of clustering is just the result of competition for the best places if those are in limited supply and natural selection favoring choosing a resting place next to another individual, or whether it is really intended by a group of conspecifics that arrange mutually and thus show, to some extent, a basic form of swarm intelligence. Clusters may also result from spatial limitation at the tips of grass blades or from the individual preference for places of lower conduction when, for example, climbing the shells of conspecifics. Up to now, no study has addressed the question whether snails compete or cooperate to find the best places to rest, or whether they act independently from one another.

### 2.3 Estivation and physiological consequences

Among the most relevant mechanisms of adaptation to heat stress is estivation (or dormancy) during the summer months. This behavioral adjustment has the consequence that snails not only restrict their activity to favorable daytime periods, but to the tolerable seasons of the year. The crucial factors for long‐term survival during estivation are to minimize water loss and to retain sufficient energy reserves, which are accomplished by metabolic depression including suppression of protein synthesis and degradation, as well as an enhancement of defense mechanisms stabilizing macromolecules (Arad, Mizrahi, Goldenberg, & Heller, 2010). Physiologically, estivation in terrestrial snails is accompanied by an accumulation of polyols (myo‐inositol, glycerol) and saccharides, which protect cells from dehydration, as in *Helix pomatia* (Nicolai et al., 2011). Cholesterol, which helps maintain membrane fluidity at elevated temperatures, is present in high concentrations (Robertson & Hazel, 1997). During estivation, polysaccharides are catalyzed first. Subsequently, proteins are the main metabolic substrate while lipids are catabolized at a low rate only (Rees & Hand, 1993). As a result of protein catabolism, urea and, to a minor extent, purine bases accumulate in the snail's body, the former correlated with mortality in *Oreohelix subrudis* after 7 months of estivation (Rees & Hand, 1993). There is also indication for a mollusk‐specific, anaerobic glycolytic pathway which generates high amounts of succinic acid during estivation (and freezing) (Livingstone, 1991; Storey, Storey, & Churchill, 2007). Estivation furthermore is characterized by a fine tuning adjustment of the molecular stress response between the stages of inactivity and arousal which is explained in detail below in the “Molecular adaptations” paragraph.

Snails that estivate attached to vegetation or other above‐ground substrates usually seal their shell apertures with epiphragms, which contain calcium carbonate. These epiphragms are air‐permeable but limit water loss when the snail is dormant. There is also evidence that epiphragms can vary in composition and thickness, whereas thick and solid epiphragms lead to increased retention of water and facilitate longer estivation periods (Machin, 1967). Considering the high water content of a snail's body, mechanisms to avoid evaporation of body water play an important role for survival during estivation. In the case of *Sphincterochila boissieri*, the soft parts of the snail contain 81% water, 11% protein, 4% ash, and minor amounts of other organic components. Schmidt‐Nielsen et al. (1971) determined the water loss from dormant *S. boissieri* in their natural habitat and showed that they lost only slightly more body weight during the day than they gained during the preceding night. They concluded that water is absorbed on the outer surface of the shell at night and evaporates during the following day. This view was supported by the fact that empty shells showed similar weight gain and loss characteristics. In this context, the texture of the shell's surface (ribbed vs. smooth) has been suggested as being responsible for the external absorption of water and the water permeability of the shell in the genus *Albinaria* (details in the Morphological adaptations section). Schmidt‐Nielsen et al. (1971) calculated the rate of water loss for *S. boissieri* to be 0.45 mg per day. Considering that an individual with a body weight of 4 g contains about 1,400 mg of water, snails could survive for four years without an external water supply under the assumption that they are able to tolerate losing half of their body water. In general, the capacity of estivating snails to retain water seems well developed. Kotsakiozi et al. (2015) showed that only one of six *Codringtonia* species (*C. helenae*) lost water during summer estivation. Also, *Cantareus apertus* individuals that were estivating for 6 months had only 14% less water than active individuals (Reuner, Brümmer, & Schill, 2008). These highly effective water retention mechanisms thus allow estivating snails to persist for a long time. However, at the same time, they prevent them from benefitting from evaporative cooling. When an active snail is challenged by a short duration heat shock, its water loss is significantly higher than in a dormant snail (as shown for *C. helenae* by Kotsakiozi et al., 2015), and in nonestivating but inactive *Xeropicta derbentina*, there is a positive linear relation of water loss to temperature between 20°C and 40°C (Petschl, 2013). Therefore, maintenance of the high body water content in active *Cantareus apertus* (Reuner et al., 2008) depends strongly on availability of water in the environment and its uptake by the snail.

Schmidt‐Nielsen et al. (1971) also investigated changes in the biochemical composition of the snails' bodies during estivation but found no major shifts and concluded that the reduced metabolic rate of dormant snails was maintained by consumption of all tissue components, as there were also no obvious energy reserves detected. Therefore, independent of possible water loss, estivating snails reduce their total mass over time. Survival during estivation not only necessitates the ability to tolerate extreme temperatures, but also to choose the optimal site for estivation (McQuaid et al., 1979).

Arousal from estivation in *Theba pisana* can either be induced by a combination of low temperatures and high humidity or by high temperatures between 40 and 42°C. In the Mediterranean, snails predominantly are active during autumn and winter when the temperature and humidity are suitable. In northern countries such as the UK, instead of estivating in the summer, *Theba pisana* hibernates in the winter to avoid the cold and is active from spring to autumn when the temperatures are high enough (Cowie, 1984a). The reason high temperatures around 40°C act as a factor triggering activity may be that the chosen estivation site was not optimal and temperatures exceed a critical, almost lethal limit. The snails are then able to potentially escape to cooler spots. The risk associated with such a strategy becomes evident when cooler conditions cannot be found by the snails and prolonged activity in the heat leads to a higher water loss, which in turn increases the risk of desiccation. Furthermore, arousal itself is thought to pose a risk of increased oxidative stress and thus requires appropriate antioxidant defense mechanisms (reviewed by Storey, 2002). Although body temperatures of *Theba pisana* estivating in closed canopy bushes were lower than those of dormant snails at exposed sites (McQuaid et al., 1979), many snails have to start estivation in exposed positions because of limitations in the number of suitable sites within reach. Therefore, in the early stages of estivation, a snail might adjust its position until it finds its final estivation spot. There are two related types of behavior that involve crawling up high to avoid high temperatures near the ground. One is the daily behavior of going down to feed at night and back up to rest during the day—this is not estivation. The other is finding a longer term resting place in which to estivate for months over a hot dry summer.

The metabolism of estivating snails is shut down to a minimum (Blazka, 1955; Nopp, 1965). In nonestivating snails, however, divergent responses have been reported, which largely accord with the external temperature. Mason (1971) investigated respiration rates at 5, 10, and 15°C in 12 gastropod species and found—with a single exception—a general trend to increasing metabolic rates with rising temperature, in accordance with Van't Hoff's rule (Van't Hoff, 1884). This was confirmed for different morphs of *Cepaea hortensis* at the same temperatures (Steigen, 1979). In a comparative study on the desert snail *Rabdotus schiedeanus* and the garden snail *Cornu aspersum*, Riddle (1977) found a general increase in oxygen consumption (as a proxy for metabolism) with increasing temperature up to 25°C. However, at high temperatures, warming may induce reduction of metabolic rates. For example, at or above 25°C, downregulation of metabolism occurs in *Cornu aspersum*, *Trigonephrus* sp., and *Rabdotus schiedeanus* (Dallas, Curtis, & Ward, 1991; Riddle, 1977); and for *Xeropicta derbentina*, the tipping point for metabolic downregulation was at 30°C (Fischbach, 2016). Decreasing metabolism at elevated temperatures may be a general phenomenon in land snails, serving to prepare them for entering estivation. As well as interspecific differences, there is intraspecific variation related to climate. For example, *Cornu aspersum* from high latitude sites had higher respiration rates and, thus, higher metabolic activity than conspecifics from lower latitudes sites which was interpreted as “metabolic cold adaptation” (Bruning et al., 2013). Oxygen consumption, frequently used as a proxy for metabolism, may define the transition from one metabolic state to another. By extrapolating from respiratory data of estivating snails, Fischbach (2016) plausibly explained the arousal of *Xeropicta derbentina* at temperatures of 15–20°C (dependent on snail size) and predicted an upper critical threshold temperature for survival of 40–42°C.

### 2.4 The thermodynamics of a snail

Even though behavioral responses of snails to insolation have been well documented, and several studies have addressed physiological responses to withstand thermal and desiccation stress, the thermodynamics of terrestrial snails are far from being well understood. A first attempt to directly quantify a snail's power (in its physical sense, *P* [W]) by microcalorimetry was conducted by Fischbach (2016) using active and estivating *Xeropicta derbentina*. Depending on snail size, *P* of an active individual amounted to 70–145 µW at 20°C and 85–110 µW at 30°C, whereas for estivating individuals, the values were 15–75 µW at 20°C and 8–55 µW at 30°C. However, it remains unclear how much convective and evaporative cooling contributes to the thermodynamics and hence survival of individuals, both in the active or the estivating stage. It is also unclear, how the oxygen supply is guaranteed during estivation and to what extent it is limited because the snail is usually sealed to the substrate and the only pathway for oxygen to reach the soft tissues would be through the calcified seal of the shell opening or the shell itself. And it is unknown whether individuals actively compete with their neighbors for best places to estivate and how they select which height to climb to as the most advantageous for their individual energy balance and efficiency of cooling by thermal convection. Is there selection pressure for climbing as high as possible? Or is climbing very high (which entails a long distance to reach the ground again for feeding) limited by the individual energy balance? Energy metabolism could be a target of selection: Artacho and Nespolo (2009) found significant directional selection on the obligatory cost of maintenance in *Cornu aspersum*. Individuals with average‐to‐reduced standard metabolic rates were supported by selection, providing support for an energetic definition of fitness. These results were corroborated by transplantation experiments with *Cornu aspersum* in Chilean field sites (Bartheld et al., 2015). In this species, artificial selection for lower metabolism early in ontogeny was associated with larger egg and adult sizes and a faster size‐specific growth rate at the expense of shell production (Czarnoleski et al., 2008). Despite open questions regarding the meaning of energy metabolism for the persistence of snails in their habitats, however, a particular aspect in their thermodynamics that has received long‐lasting attention is the transmission of heat as a function of shell morphology.

## 3 MORPHOLOGICAL ADAPTATIONS

Several morphological features are considered to influence snails' body temperature, such as the color of parts of the body (Cowie, 1990; Cowie & Jones, 1985). Mostly, however, such adaptations relate to the shell.

**Figure 2:**
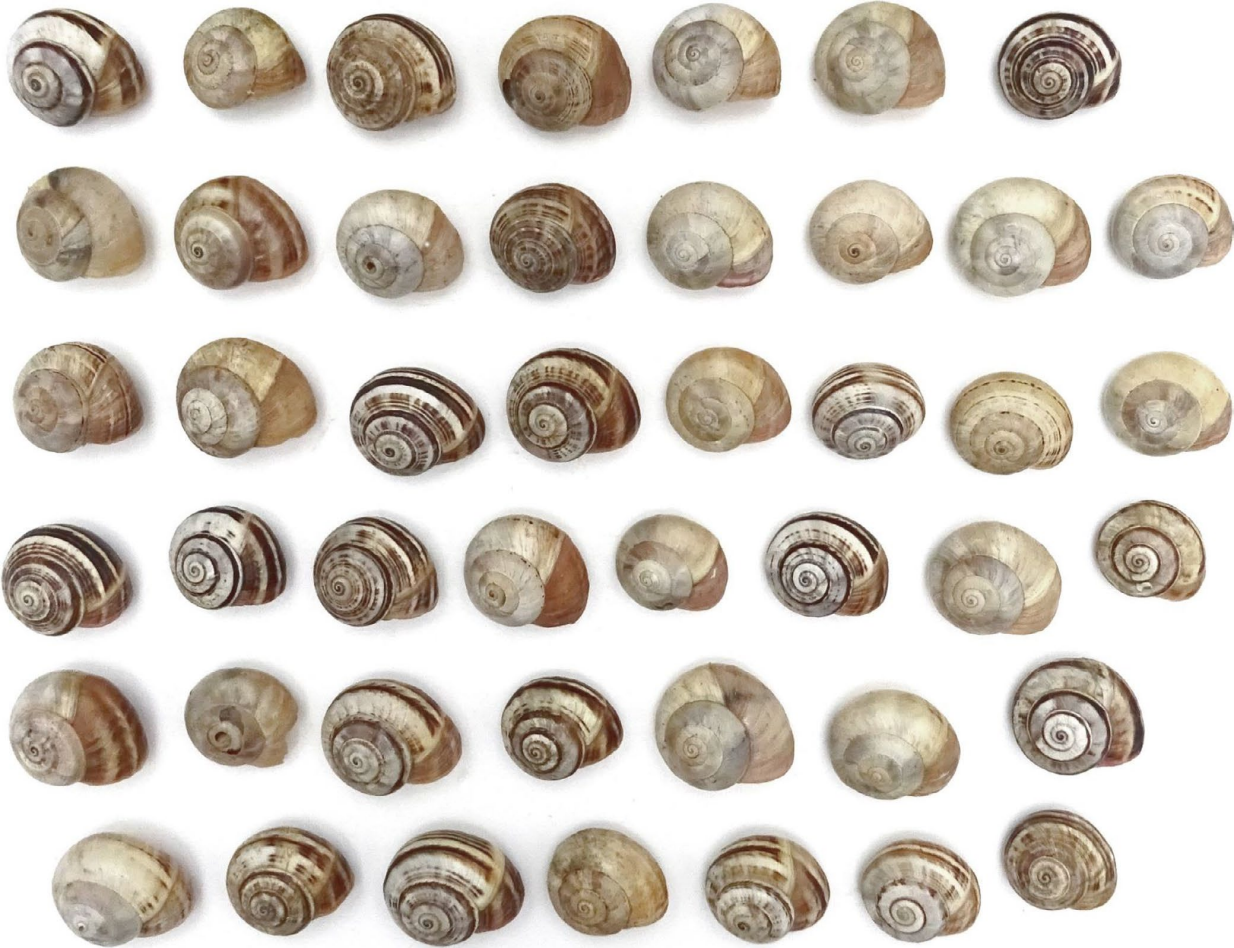
*Theba pisana* (Helicidae) displays a large variety in shell pigmentation as almost every individual has its own pattern and pigmentation intensity. Snails were generously collected from a population at the south coast of Algarve, Portugal, west of Vilamoura, by Melina Coelho da Silva, University of Algarve (photograph by Janne Burmester and Lilith Sawallich, both University of Tübingen)

The size of the shell plays an important role because of the simple mathematical fact that, at a given shape, the smaller an object the larger its surface‐to‐volume ratio. This implies that smaller snails heat up faster than larger ones, as has been experimentally shown for *Theba pisana* exposed to visual and infrared radiation in a field plot (Knigge, Di Lellis, Monsinjon, & Köhler, 2017). This study also demonstrated a more rapid temperature decline in smaller individuals under constant airflow. The surface‐to‐volume ratio is also crucial with respect to the aridity of a site. In natural populations of 28 *Albinaria* species, snails interspecifically had larger shells at Mediterranean sites at low latitudes, which were dryer, than those at more northern sites (Giokas, Páll‐Gergely, & Mettouris, 2014). Another morphological factor related to thermal and water balance is variation in shell aperture size. In dry environments, they tend to be interspecifically smaller, probably to reduce water loss (Goodfriend, 1986). To minimize water loss, two strategies have evolved in different species of the genus *Albinaria*, regarding the texture of the shells: Ribbed shells retain more water on the outer surface, whereas smooth shells exhibit lower water permeability (Giokas et al., 2014)—intermediate morphology seems not to have the same evolutionary success. Furthermore, shell orientation varies, which may be important in such snail species or individuals in which pigmentation is not evenly distributed across the shell, for example, in banded morphs of a number of species (Figure 2), as suggested by Heath (1975).

### 3.1 The role of shell pigmentation

**Figure 3:**
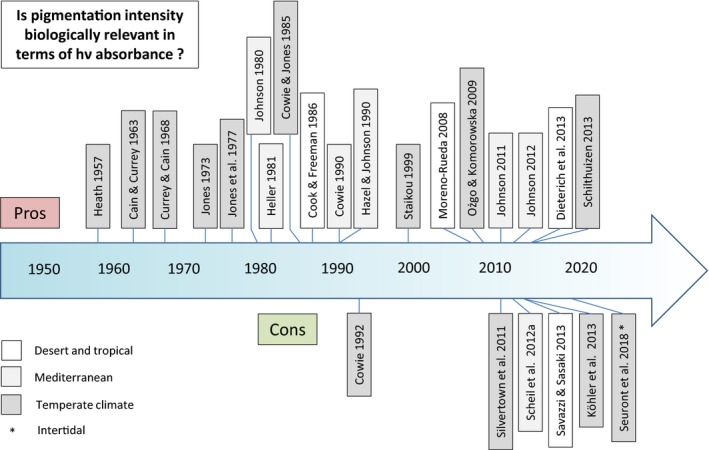
Timeline with key publications on the biological relevance of snail shell pigmentation in terms of the absorption of solar radiation. Upper part: publications providing evidence for the relevance of pigmentation for biological parameters; lower part: publications which negate such correlation. Particularly, the controversy from 2008 up to now is notable. Detailed information on these studies is provided in the text

**Figure 4:**
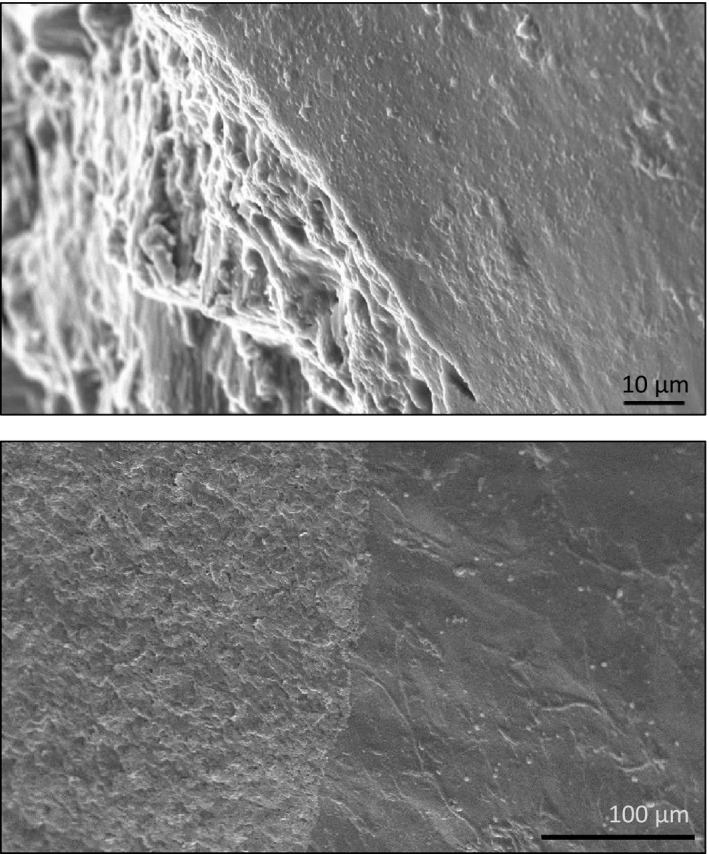
Scanning electron micrographs of the shell of *Theba pisana*. Top: artificially broken edge displaying the texture of the shell's surface (white in the visual spectrum, right part of the picture) and showing the porous character of the shell material (left). Bottom: surface of the shell at the border between the white (in vis spectrum) part (right) and a darkly pigmented stripe (left) (photograph by H.‐R.K. and Monika Meinert, by courtesy of Oliver Betz, all University of Tübingen)

Pigmentation is the best‐studied and most discussed topic in the context of morphological adaptations of snails to thermal stress. The crucial question here is whether pale morphs have a physiological and an evolutionary advantage over darker morphs in respect of heating properties in areas with high solar radiation. Despite numerous studies that related shell pigmentation to fitness parameters, Seuront et al. (2018) concluded that “the potential role of shell colour in regulating body temperature in molluscs is still poorly investigated, despite shell colour polymorphism being a common feature”. Particularly during the last two decades, several contradictory papers have been published on this topic (Figure 3), but a concluding synthesis has not been framed. In general, it is supposed that in many but by far not all ectotherms, individuals living in hotter climates are paler than those from cooler parts of the species' distribution range. In land snail species, this association has been exemplarily shown for the basic coloration (but not the banding pattern) of the shell (in *Cepaea nemoralis*, Jones, 1973; Jones, Leith, & Rawlings, 1977), the skin between the tentacles (in *Cepaea nemoralis and C. hortensis*, Cowie & Jones, 1985), and the mantle collar (in *Theba pisana*, Cowie, 1990), by using visual matches to color plates in color atlases but not considering variable or spotted pigmentation. A frequent explanation for correlations of pale coloration with hot climate is that a pale shell or body reflects sunlight and helps the animal to maintain a lower body temperature in a hot environment, whereas in cool areas, darker color is favored, as it absorbs more solar radiation and therefore enables poikilothermic animals to reach their operating temperature faster or enables homoeothermic animals to maintain their body temperature with less energy expenditure (Cowie, 1990; Johnson, 2012; Ożgo & Komorowska, 2009). In this context, however, it needs to be stressed that the emission coefficient (= absorption coefficient) of an object is largely defined by the material and only to a minor extent by its color (Eichler, Kronfeldt, & Sahm, 2005; Heuberger & Fels, 2007). The situation could even be more complicated than expected. Preliminary *SEM* investigations on the shell have revealed a rather smooth surface in contrast to the porous character of the internal shell material and suggested pigmented shell parts (bands) to be associated with slight modifications in the texture of the surface (Figure 4) but it remains unclear what all this means for the shell's water penetrability and, thus, evaporative cooling. Nevertheless, the majority of studies consider shell pigmentation to be biologically and evolutionarily relevant.

In one of the first studies on this subject, Heath (1975) proclaimed a pigmentation effect in different shell color morphs of *Cepaea nemoralis* on their natural or an artificial interior: mercury‐filled shells and mercury‐topped up living, retracted snails were exposed to sunlight either unmodified or with artificially blackened shells. The darker morphs, in both unmodified cases, were significantly warmer than the lighter ones, and they showed an additional increase in temperature when painted black. Jones (1973), who also conducted heating experiments with *Cepaea*, found dark snails to reach a higher temperature than pale ones in more than 90% of cases. Also in *Theba pisana*, fully banded individuals exposed to full sunlight heated up more rapidly than did unbanded snails (Hazel & Johnson, 1990). In another experiment on the thermal capacities of differently pigmented snail shells, Cook and Freeman (1986) used a microscope bulb for artificial illumination and the results were in the same direction. Even though all these studies used artificial settings and techniques of contact measurement to determine temperature deltas, the constancy of findings implies fundamental importance of shell pigmentation for the thermodynamics of snails, at least in situations of strong irradiation. Even morph frequencies in subfossil samples of *Cepaea nemoralis* from Britain could be related to prehistoric climate data in agreement with present‐day evidence (Currey & Cain, 1968). In the species *Sphincterochila boissieri*,* Sphincterochila candidissima*, and *Trochoidea seetzeni*, in which almost exclusively pale morphs exist, it is evident that their light color can be regarded as an important factor in the heat balance of those snails. The measured reflectance of solar radiation is high: For *Trochoidea seetzeni*, it is about 80% (Yom‐Tov, 1971), and for *Sphincterochila boissieri*, reflectance totals well over 90% (Schmidt‐Nielsen et al., 1971; Yom‐Tov, 1971). Therefore, being white seems to be highly advantageous under high solar radiation since *Sphincterochila candidissima* individuals have been reported to lose considerably more water during estivation when artificially painted black (Moreno‐Rueda, 2008). Following the observations of Jones et al. (1977) who found that *Cepaea* populations from hotter regions tend to display higher frequencies of pale shells, Cowie (1990) focused on the pigmentation of soft tissue, the mantle collar. The color intensity of the mantle collar of *Theba pisana* individuals was scored and plotted versus the mean daily maximum of the hottest month in European locations, Northern Africa, the Middle East, Australia, and California. The result showed a significant relationship between among‐population variation and the mean daily temperature of the hottest month and revealed a strong association between pale mantle collar color and hot climate. Cowie (1990) concluded that there is climatic selection presumably favoring pale color in hot environments, even though he admitted that the mantle collar is not the body part that is most exposed to sunlight. Another study of Staikou (1999) investigated the activity levels of different *Cepaea vindobonensis* morphs and their resistance to desiccation. In the field, adult dark‐banded morphs were found to be less active and mobile in general than faint‐banded snails and juveniles of both morphs during sunny mornings or under severe heat load. Also, an intraspecific difference in regard of the water loss was detected between faint‐ and dark‐banded morphs with faint‐banded morphs showing no change in relative body water after desiccation. In addition to all the correlative studies cited before, this finding also supports the opinion that lighter‐colored morphs are better adapted to arid conditions and, therefore, Staikou (1999) infers that a lighter shell color in *Cepaea vindobonensis* may be a major, evolutionarily favored adaptation enabling these snails to cope with heat stress or water loss. However, approaches to quantify the degree shell pigmentation modifies the shell's emission coefficient and, therefore, shell temperature and, eventually, the internal body temperature under natural conditions in the habitat, and to decide whether such impact is biologically relevant in terms of advantages or disadvantages for selection, require elaborate and still missing studies that involve a comparison of natural pigmentation with artificial coloration of different intensity and that combine state‐of‐the‐art technology in noncontact thermometry with long‐term field experiments.

### 3.2 Evolutionary aspects of shell coloration variability

At the beginning of this decade, a large citizen science project (“*Evolution MegaLab*”) tested the hypothesis that global climate change‐exerted increase in environmental temperatures in Europe had caused selection in favor of the lightest morphs in *Cepaea nemoralis*, which have a basic yellow coloration (Silvertown et al., 2011). This project did not reveal any increase in the frequency of yellow shells with time but rather a general unexpected decrease in the frequency of unbanded shells (independent of the basic coloration which can be yellow, pink, or brown) and an increase in the “mid‐banded” morph (with fewer than the maximum of five bands). The study, indeed, confirmed the known historical geographic cline of the yellow‐colored phenotype of *Cepaea nemoralis* (Jones et al., 1977)—the one with the supposedly highest albedo—and its rather static persistence until 2009. The frequency of the “yellow” morph did not rise despite an average increase of 1.3°C in Europe during the 20th century with a particularly steep ascending slope between 1990 and 2009 (European Environment Agency, 2018). Even contrary to the assumption that increasing temperature is selecting for a low number of dark bands in this species, the frequency of “unbanded” morphs decreased by about 10% within 15–20 generations with ongoing global warming in Europe (Silvertown et al., 2011). Furthermore, the observed increase in the “mid‐banded” phenotypes at the cost of those with more bands was particularly not occurring in those regions in which temperatures having increased the most. Silvertown et al. (2011) therefore concluded from this finding that selective factors other than climate change‐induced temperature increase, for example, changing predation pressure and habitat change with effects on microclimate might have prevailed. Despite its novelty at that time, however, this citizen science paper has been critically assessed for its potential liability for observer bias, even by the authors themselves.

When relating morph frequencies to habitat types throughout Europe, Silvertown et al. (2011) found banded *Cepaea nemoralis* to be less frequent in dunes than in other habitats, independent of the mean temperature in these areas. In a more detailed follow‐up study using the *Evolution MegaLab* database, furthermore, Cameron and Cook (2012) found the association of habitat type and coloration, including the frequency of banded individuals of *Cepaea nemoralis* to be inconsistent in Europe. The correlation became clear for Southern England and the north of France where yellow individuals exhibited a higher frequency in open habitats than in woods. This was not the case for other regions. The strongest association between banding and the habitat was also reported for Southern England, however with higher frequencies of unbanded individuals in woods that in other habitats which has led to the conclusion that banding is probably not the most important character selection has acted upon (Cameron & Cook, 2012). Unbanded morphs from woods, however, are predominantly nonyellow (i.e., pink, brown), and thus, darker individuals have been allocated to woodland rather than to open habitats (e.g., Cain & Sheppard, 1954). In a study on the evolution of *Cepaea nemoralis* morphs in the course of land reclamation, Schilthuizen (2013) also reported populations in shaded habitats to have evolved toward darker shells than those in the adjacent open habitats. Similar findings have been reported for *Theba pisana* in Israel (Heller, 1981) and for *Cepaea vindobonensis* in Poland (Ożgo & Komorowska, 2009). All these observations and similar findings have led to the common interpretation that morph frequencies in these and other land snail species are delimited by crypsis and/or apostatic selection by visual predation by birds or rodents (Allen, 2004; Bond, 2007; Cain & Currey, 1963; Cain & Sheppard, 1954; Clarke, 1969; Cook, 2005; Endler, 1978; Heller & Gadot, 1984; Moreno‐Rueda, 2009; Punzalan, Rodd, & Hughes, 2005; Rosin, Olborska, Surmacki, & Tryjanowski, 2011), but, to a controversially discussed degree, also by the respective microclimate of the habitat resulting in climatic selection (Hazel & Johnson, 1990; Heller, 1981; Johnson, 1980; Lamotte, 1959; Ożgo, 2011; Schilthuizen, 2013). Indeed, a meta‐analysis of the dataset of Cain and Sheppard (1954) and 18 other datasets confirmed the prevalence of nonyellow unbandeds in woods and yellow bandeds in open habitats (Cook, 2008). Nevertheless, Cook (2008) stated that visual predation is not incontrovertible as nonvisible differences in fitness could also be involved in selection. In a study on the crypsis of *Cepaea nemoralis* individuals as seen by birds, Surmacki, Ożarowska‐Nowicka, and Rosin (2013) found dry vegetation, common in open landscapes and also dunes, to be the most cryptic habitat and, within it, yellow unbanded individuals to be most cryptic. Generally, banded morphs were more conspicuous to a bird's sight than unbanded ones which contrasts with the real situation in which yellow banded individuals are most frequent in open habitats. Therefore, these data cannot exclusively explain the high prevalence of the yellow and banded morph of *Cepaea nemoralis* in open habitats by avian predation, and potential relevance of thermodynamic aspects for the selection of differently pigmented phenotypes cannot be excluded, even though the analysis of big datasets failed to give evidence for the latter at least in *Cepea nemoralis*. Future studies that aim to separate the evolutionary meaning of climate from crypsis thus need to quantify pigmentation intensity in a more elaborate way than by banding pattern classification, particularly in species with large individual variation (e.g., *Theba pisana*), and combine these analyses with GIS‐based methods that allow to quantify the extent of vegetation cover and advanced statistics such as multiple regression modeling or random forest algorithms.

### 3.3 Critical comments on the pigmentation effect

**Figure 5:**
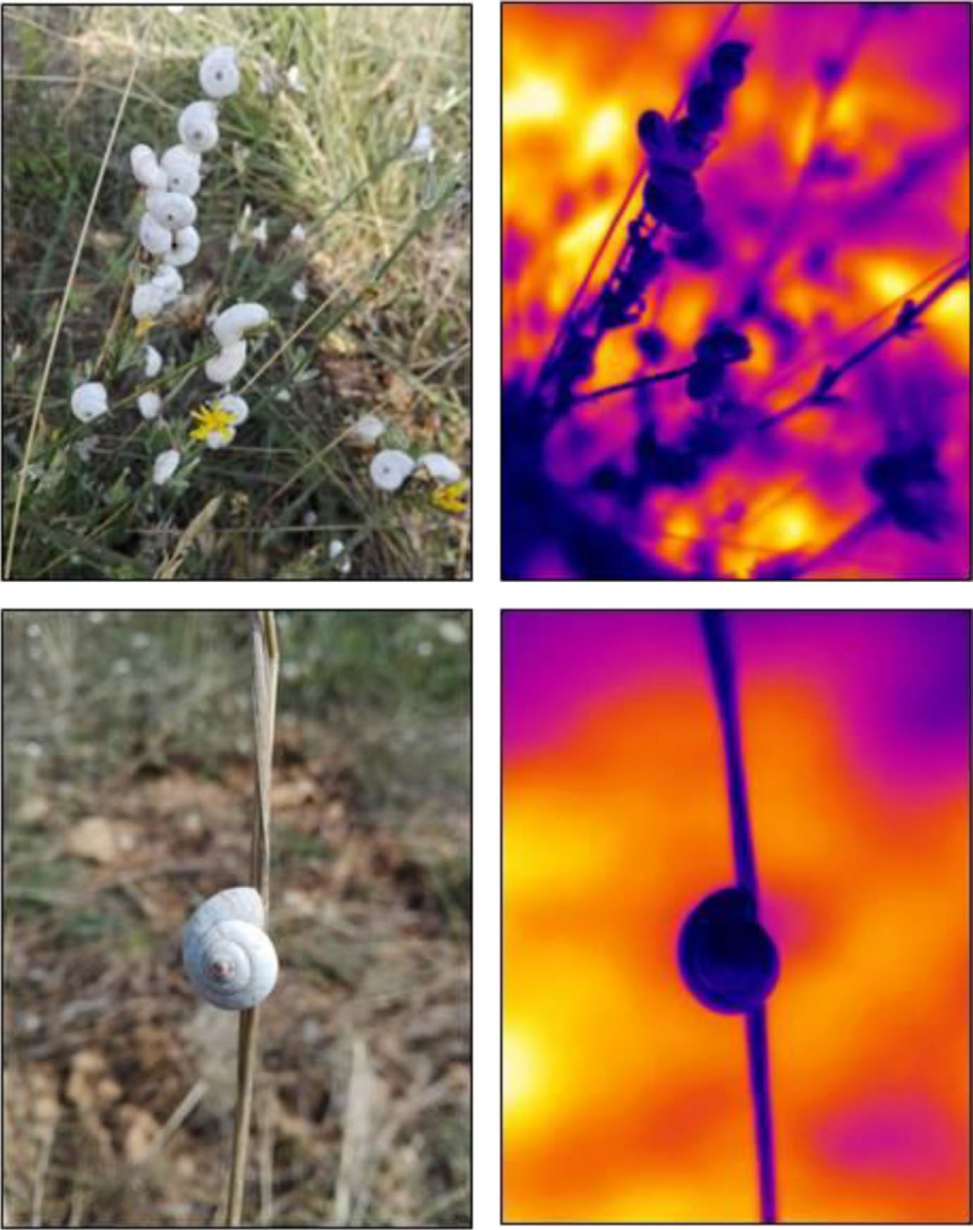
Thermographic infrared pictures (right) of *Xeropicta derbentina* (Hygromiidae) in their natural habitat (pictures taken in the visual spectrum on the left) north of Lac d'Esparron, Alpes‐de‐Haute‐Provence, France (photographs by H.‐R.K.). Climbing the stalks leads to a much lower temperature of the shells (displayed in blue and black) than of the ground (white, yellow, orange, and purple). The pictures at the bottom show that, despite illuminated from above (left), the shell temperature of the part facing the ground is a little higher (right, blue) than that of the upturned part (right, black). This substantiates the assumption that the snails are decisively heated up by a vertical convectional flow of hot air and/or irradiation reflected at the soil surface

Further studies picked up the topic of possible evolutionary advantages of pale shell morphs under solar radiation in concern of heating. For example, Scheil, Gärtner, and Köhler (2012) did not find any differences, neither in absolute warming or heat loss nor in warming or heat loss kinetics of empty shells of light and dark‐banded *Theba pisana* morphs under highly reproducible though artificial conditions (full spectrum bulb) using thermography. Despite the high potential of infrared thermography (Figure 5) in mollukcan thermophysiology research (Seuront et al., 2018), the use of empty shells in the study of Scheil, Gärtner, et al. (2012) did not match the situation in the living animal and allows the transfer of heat to the air‐filled interior of the shell. Also questioning the expectation that darker morphs in general absorb more energy from solar radiation, Gunn (1942) had stated that the colors humans are able to perceive are generated by reflecting wavelengths within the visible spectrum, but do not take into account other wavelengths, for example, near infrared, an important factor in thermal energy transfer. In this context, an interesting finding was reported by Savazzi and Sasaki (2013), as they observed that visible differences between differently colored snail shell morphs disappeared when the animals were illuminated exclusively by near‐infrared light. The importance of infrared light for the heating of snails, however, does not exclude the contribution of wavelengths in the visual spectrum to energy transfer. In the eulittoral species *Littorina saxatilis*, the ratio between body and substratum temperatures did not vary among four morphs (from dark to light individuals) in natural populations at low tide, when the snails apparently experienced terrestrial conditions (Seuront et al., 2018) but, in this environment, thermodynamics are known to be dominated by complex interactions of physical factors with the animals' size, morphology, and interactions with neighbors, which may create feedback loops between abiotic and biotic control, as shown for intertidal mussels (Helmuth, 1998, 1999).

Also in particular terrestrial habitats, shell pigmentation does not necessarily have to be a decisive feature. No thermobehavioral differences were found between differently colored *Theba pisana* morphs from Wales (Cowie, 1992a). This suggests that differences in the thermal capacity of different morphs have not been biologically relevant enough to select for particular behavioral strategies in differently pigmented morphs of this species. But this finding does not generally contradict the meaning of shell color for snail thermodynamics as already Cowie (1992a) explained his observation with the location of the investigated population and the cool climate at the northern limit of the species' distribution. In such areas, the need of darker morphs to behaviorally or physiologically adapt to avoid over‐heating may not be necessary because none of the morphs suffers from the rather low ambient temperatures. Therefore, a more detailed picture on the association between the coloration of different morphs and their behavioral and physiological capacities, however, would require records of possible heat‐induced damage to tissues, measurements of energy allocation and possible concomitant constraints which, on the long run, may lead to differences in behavior and physiology between morphs.

### 3.4 Is there also a benefit of being dark?

In contrast to findings that support climatic selection of pale morphs in hot areas (Cowie, 1990; Cowie & Jones, 1985; Jones et al., 1977), in some species and areas, darker‐pigmented morphs occur in relatively high numbers. In cooler places, the greater ability of darker shells and bodies to absorb radiation and thus allow adequate activity, feeding, and growth may favor more intense pigmentation (Cowie & Jones, 1985; Heath, 1975; Jones, 1973). However, also in some hot areas rather dark individuals are found (e.g., Scheil, Scheil, et al., 2012). Thus, it is reasonable to propose that darker pigmentation may pose also benefits possibly trading‐off thermal disadvantages. The dark color of the shells is caused by melanin, which is known to act as an effective antioxidant by absorbing free radicals generated by oxidation of primary metabolites. In a study on transcriptomics in *Cepaea*, Kerkvliet, de Boer, Schilthuizen, and Kraaijeveld (2017) suggested metallothionein genes to be involved in the formation of the banding pattern. Metallothionein is thought to inhibit melanin production following oxidative stress (Sasaki et al., 2004) and, therefore, may in turn decrease the pigmentation intensity of the shell. Since elevated temperature can cause oxidative stress, one may speculate, however, that rather a high melanin level could be advantageous. This hypothesis was tested by Scheil, Scheil, et al. (2012) using the lipid peroxidation level as a biomarker for heat impact and, concomitantly, oxidative stress in differently colored *Theba pisana* morphs. However, the study did not reveal any effect of shell color on the lipid peroxidation level, and thus, it can be concluded that dark‐banded morphs are not able to limit the effects of oxidative stress to a greater extent than pale morphs. If there was an advantage of being dark, it should be attributed to other factors but fighting lipid peroxidation, for example, escaping predation, meeting requirements of the microhabitat (Scheil, Scheil, et al., 2012), or increasing immunological properties. In this context, it was shown that darker morphs of *Theba pisana* and *Cernuella virgata* were significantly less infested by nematodes than pale conspecifics (Cabaret, 1983, 1988; Scheil, Hilsmann, Triebskorn, & Köhler, 2014) and that climatic conditions influence parasite frequency in snails (Morley & Lewis, 2008) Furthermore, dark individuals of *Cepaea hortensis*, *Cornu aspersum*, and *Theba pisana* have been shown to be less sensitive to hemolymph withdrawal (Scheil, Hilsmann, Triebskorn, & Köhler, 2013). Thus, it is surely worth investigating possible links between parasite/infection tolerance, climate, and snail pigmentation in the future.

### 3.5 The maintenance of variation in shell pigmentation

Despite the high number of studies on shell pigmentation, the evolutionary role of shell polymorphisms, with respect to thermal aspects, is still not fully understood. It is commonly accepted that in some habitats and in some species, solar radiation may be a dominant environmental factor, in other places or species it may not. A plausible mechanism to maintain polymorphism at boundaries between exposed and sheltered habitats has been reported by Johnson (1981) for *Theba pisana*. Mark–recapture studies revealed variation largely to result from net movement from and to the sheltered habitat and differential habitat choice in banded and unbanded individuals. This mechanism, however, cannot explain the maintenance of variation in uniformly hot habitats. Although to date there are not many studies statistically linking morph frequencies of snails to environmental parameters such as heat, three of them (Johnson, 2011, 2012; Köhler et al., 2013) have identified moderately elevated temperatures acting on free‐living populations of *Theba pisana* in the middle of their lifetime as a key element for the variation in shell coloration. The studies of Johnson (2011, 2012) found hot summer conditions to increase the frequency of unbanded morphs in *T*heba *pisana*, attributing this phenomenon to climate selection. The very same phenomenon was observed by Dieterich et al. (2013) for X*eropicta derbentina*; however, it remains to be clarified by further studies whether this is due to selection or shell bleaching, the latter eventually resulting from the inhibition of the melanin synthesis via oxidative stress/metallothionein‐induced stress proteins (Hsp70) as it has been observed for human or mouse melanocytes (Hoshino et al., 2010; Sasaki et al., 2004). The banding of shells has a well‐characterized genetic background (Cowie, 1984b; Jones et al., 1977; Murray, 1975) but suppression of pigmented bands in *Cepaea nemoralis* by expression of the hyalozonate gene is a long‐known phenomenon by which the phenotype of the shell can be modified (Cain, Sheppard, & King, 1968). In contrast to a directed selection (Johnson, 2011, 2012), Köhler et al. (2013) who used a dataset of Cowie (1992b) showed high winter and spring temperatures to be associated with high variation in morph frequencies but not with the frequency of a particular morph. Also, a study on the phenotypic disequilibrium in a large dataset obtained for *Cepaea nemoralis* suggested that selection has favored combinations of common morphs, whichever they were (Cook, 2005). Thus, a maintenance of shell color polymorphism in some helicoid snail populations for decades can be the result of changes in the direction of selection between different places and times (as interpreted by Cook, 2005; Johnson, 2011; Johnson, 2012) or of epigenetic nondirectional changes within phenotypic plasticity (as interpreted by Köhler et al., 2009; Köhler et al., 2013). Both selection of varying directions and phenotypic plasticity could lead, in combination with migration, to prolonged polymorphism within a population (Cook, 2005; Köhler et al., 2013). Furthermore, selection pressure by predation, drift, founder effects and differentiation in refugia leading to area effects (Cain & Currey, 1963) contribute to maintain polymorphism in populations (Carter, 1967; Cook, 2005; Cook & Pettitt, 1998; Davison & Clarke, 2000; Jones et al., 1977). Despite early accounts of apostatic selection (Clarke, 1962, 1969), frequency‐independent forces are regarded a more plausible explanation for the persistence of polymorphism (Cain & Currey, 1963; Carter, 1967; Cook & Pettitt, 1998) which may last over long time (Cook, Cowie, & Jones, 1999; Cowie, 1984b; Cowie & Jones, 1998). Nevertheless, the interplay of mechanisms remains unresolved (Cook, 2013).

Regarding thermal aspects, one has to conclude that there is probably not a single, universally valid answer to the question, to what extent dark pigmentation is disadvantageous or advantageous under insolation—this rather seems to depend on the intensity of pigmentation in different morphs (and, thus, eventually on the degree of disparity in the level of thermal capacities between morphs) which may vary among species and populations, and on the climate (i.e., the biological meaning of insolation under the given circumstances in either arid or temperate climate, relative to other selective factors such as, e.g., predation). The intensity and duration of solar radiation at which differences in shell coloration become relevant for selective processes, therefore, remains to be investigated—particularly in view and in expectation of global warming. Hence, in some habitats, the integrity of animal physiology is likely to be buffered against fluctuations in temperature, even though small differences in internal temperature may decide between life and death when temperatures reach upper physiological limits. The relevance of shell pigmentation for selective heat death in the field under conditions of intense radiation has been shown for sand dune populations of *Cepaea nemoralis* (Richardson, 1974, 1979). Apart from mortality, differences in thermal properties of snails could lead to evolutionarily relevant changes in behavioral or life cycle traits, including activity patterns, growth rate, fecundity, or mating ability (Staikou, 1999).

## 4 CELLULAR ADAPTATIONS

In contrast to the rather extensive data availability which exists on physiological, morphological, and—as shown later—molecular responses to solar radiation and its evolutionary consequences on shell pigmentation, the number of histological studies in this context is limited. Only few papers report on cellular responses of land snails to hot and dry conditions although action on the cellular level, namely the secretion of mucus, is among the first‐line defenses when snails experience a sudden increase in heat. In general, mucus production and secretion can contribute to up to 23% (related to ingestion) in the energy budget of snails (Davies, Hawkins, & Jones, 1990) and thus represent one of the most important processes in snails' physiology, even though snail mucus contains mostly water. Snail's mucus is a secretion product of different mucous cells in the skin, and mucus secretion can be used to cool the body via evaporation of its aqueous content (Dittbrenner et al., 2009). Slugs react to environmental stress in general (and to artificial irritation) with increased mucus production, but this response is always accompanied by the risk of dehydration because of the high water content of the extruded mucus (Triebskorn, Christensen, & Heim, 1998). Not only the quantity of mucus but also its quality can differ based on the activity of different mucocyte types, which produce mucus of different chemical composition (Triebskorn et al., 1998).

**Figure 6:**
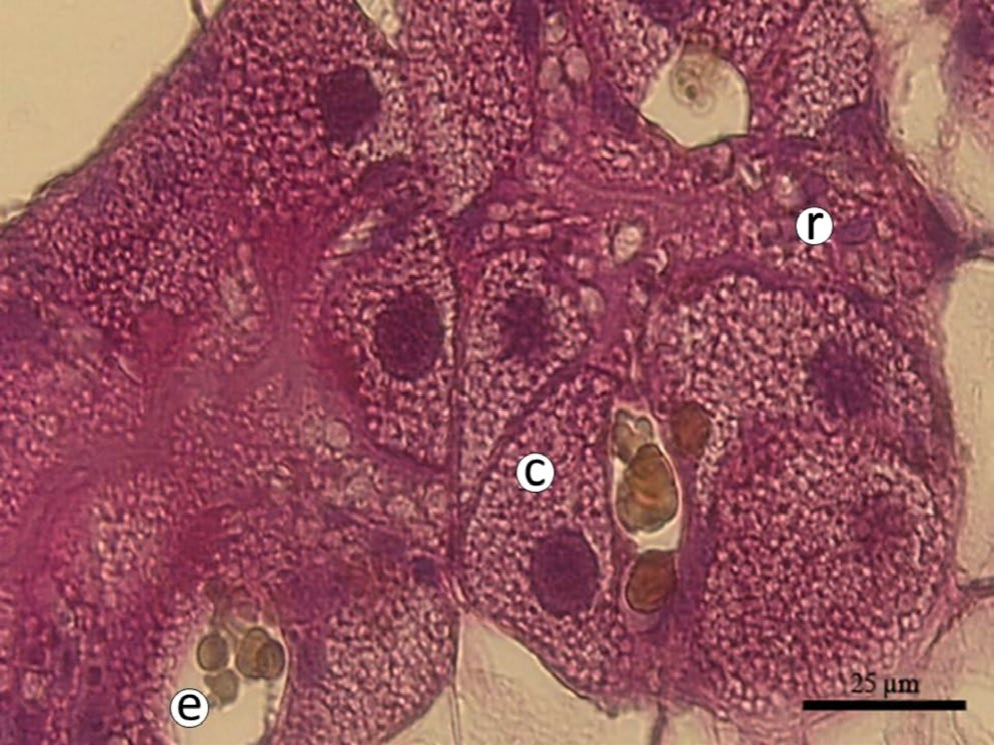
Section of hepatopancreatic tissue of *Xeropicta derbentina* (Hygromiidae) displaying hypertrophic calcium cells after exposure to 45°C for 8 hr. c: calcium cell (= crypt cell, basophilic cell), e: excretion cell, r: resorptive cell (= digestive cell) (photograph by Nils Dittbrenner, University of Tübingen)

In a histopathological study, Dittbrenner et al. (2009) investigated the influence of increased temperature on mucocytes in the foot of individuals of *Theba pisana*, *Cernuella virgata*, and two *Xeropicta derbentina* populations. They showed mucus secretion increased with increasing external temperature, and mucocytes displayed hypertrophy, which is assumed to be the reason for the ability of the snails to thermoregulate by evaporative cooling. This form of physiological adaptation, however, does not work for estivating snails, as they are sealed in their shells and cannot refill their water storage and therefore have to be careful with their reserves; nor does it work for snails in extremely arid habitats, as these animals rely on their mechanisms of water retention, which do not allow any waste of fluids. Dittbrenner et al. (2009) also investigated calcium cells in the digestive gland, which play an important role in osmoregulation of pulmonates. They showed that the total percentage of calcium cells in the digestive gland rises with increasing exposure temperature. They also found that the most heat‐tolerant snails in their experiment (*Xeropicta derbentina*) already possessed a higher abundance of calcium cells at lower temperatures (25°C) than the other, less heat‐tolerant species. *Xeropicta derbentina* was also capable of responding to heat stress very quickly by increasing the size (hypertrophy) and number (hyperplasia) of calcium cells in their digestive gland. Hypertrophy and hyperplasia of calcium cells in turn led to a concomitant loss of hepatopancreatic digestive cells, which is consistent with findings that mollusks generally lose digestive cells in response to environmental stress (Zaldibar, Cancio, & Marigómez, 2007). In addition, Troschinski, Di Lellis, et al. (2014) showed that calcium cells in the hepatopancreas of 7*derbentina* were more heat‐resistant than digestive cells in the same individuals when they were exposed to external temperatures of up to 52°C. Therefore, the difference in heat tolerance of the snails in this study seems to be connected to the ability for rapid and extreme proliferation of calcium cells in the digestive gland and can thus be regarded as a process of cellular adaptation to heat stress. The aforementioned study was supported by Scheil, Köhler, and Triebskorn (2011), who compared differences in the heat tolerances of *Xeropicta derbentina* and *Theba pisana*, probably caused by the different ability of calcium cells in the digestive gland to proliferate. They also tested whether the snails possessed the potential to recover after heat exposure. During heat exposure, calcium cells were proliferating very actively. Particularly, the more heat‐tolerant *Xeropicta derbentina* individuals showed a significant increase in the absolute number of calcium cells after experiencing two hours of heat, which may be interpreted as a prompt attempt to regulate thermal stress and, possibly, to replace already heat‐impaired cells. The higher number of calcium cells in *Xeropicta derbentina* at all time points, in general, seems to be a consistent adaptation to the higher temperatures this species encounters in its natural habitat. This response seems to be rather nontransient, as *Xeropicta derbentina* did not change the structure of its hepatopancreatic calcium cells upon recovery from heat for several hours (Scheil et al., 2011). Whereas *Xeropicta derbentina* could respond to extreme temperatures with hypertrophy and hyperplasia of calcium cells (Figure 6), no such adaptations were found in *Theba pisana*. However, as *Theba pisana* nonetheless displays a rather high tolerance to heat compared to most terrestrial snail species, it can be assumed that it has different, as yet unknown mechanisms that protect cellular function during thermal stress.

Cellular adaptations that influence the color of the shell and thus may contribute to the discussion on the evolutionary role of this character are almost unknown. Emberton (1963) found the density of melanocytes in the mantle of *Cepaea hortensis* and *Cepaea nemoralis* to be correlated with the darkness of the shell's lip and, possibly, its banding pattern. A mechanistic relationship with a metallothionein‐based reduction of melanin production (Sasaki et al., 2004) mentioned above, however, remains speculative.

## 5 MOLECULAR ADAPTATIONS

It is almost impossible to separate molecular adaptations from cellular or physiological processes because of the complex interactions within biological systems, particularly when using a holistic approach as in this review. For editorial reasons, the systemic aspects of estivation in response to arid conditions have been included in the “physiology” section while the following paragraphs focus specifically on the mechanisms fighting proteotoxic and oxidative stress.

### 5.1 The molecular heat stress and desiccation response

A major component of the molecular stress response to heat is the induction of heat‐shock proteins (Hsps), molecular chaperones assisting in protein folding and assembly. With the exception of polar ice fish, Hsps are found in almost any higher organism. Induction of Hsps is not only restricted to heat stress but proteotoxic stress in general, including proteotoxicity exerted by chemicals, osmotic stress, and desiccation (Arad et al., 2010; Mizrahi et al., 2010). According to their molecular weight and their degree of homology, Hsps can be subdivided into distinct protein families among which Hsp60, Hsp70, and Hsp90 are best characterized. The major families in turn include both constitutively expressed as well as stress‐inducible protein isoforms (Storey & Storey, 2011).

**Figure 7:**
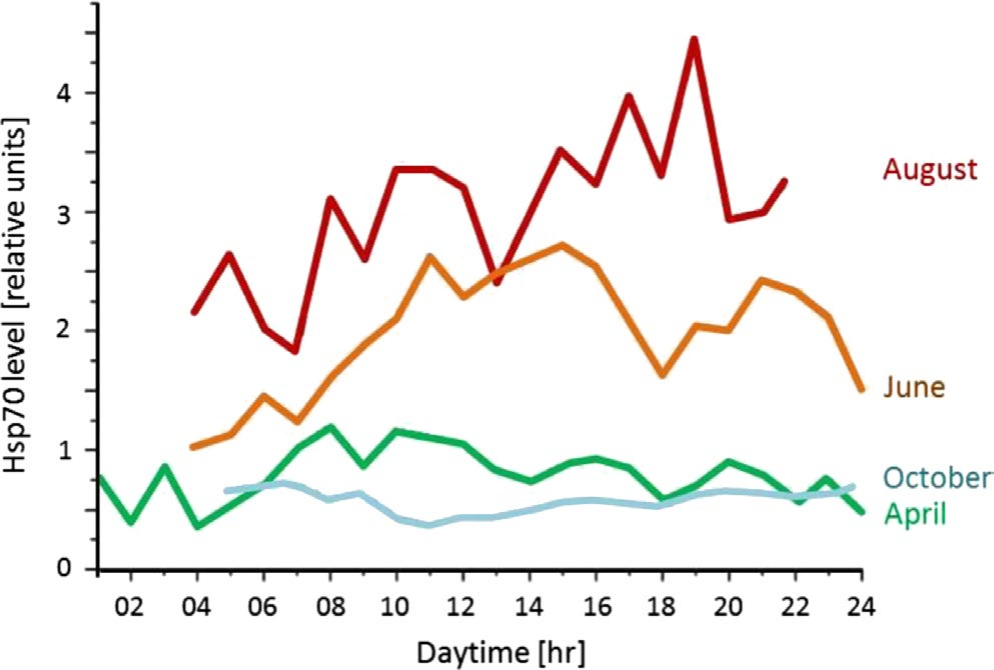
Daily Hsp70 level courses in different months, according to the data obtained by Dieterich et al. (2015) for *Xeropicta derbentina* (Hygromiidae) in its natural habitat, a meadow near Modène, Vaucluse, France. Sampling took place on 18 April, 13 June, 30 August, and 17 October 2011

Concerning heat stress, Hsp70 and Hsp90 have been of particular interest in snails. Köhler et al. (2009) compared four different snail populations (two of *Xeropicta derbentina*, *Cernuella virgata*, *and Theba pisana*) with respect to their Hsp response to elevated temperatures, also in connection with their shell pigmentation as a possible confounding factor. After 8 hr of elevated temperatures, the different populations showed different Hsp response patterns. Whereas in *Cernuella virgata*, low levels of both Hsp70 and Hsp90 were found, and in the uniformly white *Xeropicta derbentina* population, just the Hsp70 level was elevated and Hsp90 remained low. In *Theba pisana* as well as in the *Xeropicta derbentina* with an intermediate variation in shell pigmentation, Hsp70 was induced at an intermediate level, whereas Hsp90 was maintained at a low level. Although the snails' Hsp system only responded to rather high temperatures, the similarity between all four populations seems to symbolize the importance of particularly Hsp70 in their reaction to heat stress, whereas Hsp90 appears not to be affected. In a study on the proteotoxicity of heat and pesticides to Xeropicta derbentina, Krais (2008) also found the Hsp70 level to be related to heat (with 43–45°C for 8 hr resulting in a maximum Hsp70 level; in contrast to 38–40°C as reported by Dieterich et al., 2015) rather than to the two pesticides chlorpyrifos or carbaryl. Hsp90 did respond to all investigated stressors only to a minor extent. In *Theba pisana*, both the *hsp70*‐ and *hsp90*‐mRNA levels increased in response to thermal stress, but the *hsp70*‐mRNA level increase was greater and its induction continued up to higher temperatures, compared to *hsp90* (Mizrahi, Goldenberg, Heller, & Arad, 2016). These findings have also been supported by Mizrahi et al. (2010) stating that there are other interspecific studies demonstrating particularly higher endogenous levels of Hsp70 in more heat‐sensitive species compared to heat‐resistant ones. Furthermore, the capacity to induce Hsp70 depends on the physiological status of an individual, as warm‐adapted *Theba pisana* showed maximal *hsp70*‐mRNA induction at higher temperatures and also higher upper thermal limits of hsp mRNA synthesis (Mizrahi, Goldenberg, Heller, & Arad, 2015). Thus, different species or populations seem to differ in their strategies to cope with heat stress at the molecular level, a finding that has also been confirmed by Di Lellis et al. (2014). Concerning temperature‐correlated seasonal variability of Hsp70 levels in *Xeropicta derbentina* in the field, Dieterich et al. (2013) found the Hsp70 level to be positively correlated with shell surface temperature in April, June, and August only, while a negative correlation was observed in October. The latter phenomenon is especially interesting as ambient temperatures in April did not differ from those in October, and the authors concluded that this is a consequence of snails having lost their ability to react to heat stress adequately in October. They suggest three possible reasons for this: first, an energetic trade‐off between maintaining stress response capacity and reproduction, in which the focus is shifted to reproduction, particularly in older individuals; second, as described by Nakano and Iwama (2002), Tomanek (2002) and Scheil et al. (2011) before, an overwhelmed or exhausted stress response system resulting from constant heat exposure during summer in combination with a shortage in energy supply; and third, a reduced metabolism as a result of snails having entered the estivation phase (Storey, 2002) at the end of summer in the Mediterranean. According to Kiss, Labaune, Magnin, and Aubry (2005), *Xeropicta derbentina* can shift from an annual to a biennial life cycle when conditions become unfavorable, and Dieterich et al. (2013) observed some of their populations having already become dormant in October. These authors also analyzed daily Hsp70 level courses in different seasons (Figure 7). In general, they found Hsp70 levels to follow the rise of ambient temperatures during the day. In April, when temperatures were below the Hsp70 upregulation threshold temperature of 30°C (Köhler et al., 2009), this increase in Hsp70 levels was rather limited. However, in June, it was more prominent, lasting to the early afternoon and falling with sunset, followed by a further slight increase during the night. Dieterich et al. (2013) concluded that this further Hsp70 elevation at night probably was a consequence of the snails' nocturnal activity period starting when temperatures sank a few hours after sunset. During this nocturnal activity period, snails can often be observed moving and foraging, and they are likely to be in a phase in which they balance their internal milieu and produce new proteins (Herreid & Clyde, 1977; Riddle, 1981; Storey, 2002; Umezurike & Iheanacho, 1983). In August, Dieterich et al. (2013) found the daily course of the Hsp70 level to remain at a high level albeit with high standard deviations, which probably result from an interaction of seasonally caused high ambient temperature and energy‐depriving maturation of gonads, furthering the collapse of the stress protein system in some individuals. During October, the maximum temperature recorded was 23°C, and only a constitutive level of Hsp70 was observed, corresponding well with the fact that this temperature is insufficiently high to induce Hsp70 (Köhler et al., 2009).

Scheil et al. (2011) showed that Hsp70 levels display some ability to recover from thermal stress within a short period of time. Within some hours of experimental recovery from heat stress, the Hsp70 level adjusted to normal in *Xeropicta derbentina* while *Theba pisana* did not show any sign of recovery. The authors suspected that the snails, which had been collected in the field in early summer, had already experienced heat stress prior to the experiment, which led to an excessive demand on the stress protein system in the case of the more temperature‐sensitive species *Theba pisana*. For *Xeropicta derbentina*, it was assumed that, regarding its Hsp70 levels, it could recover even from severe heat stress if no energy shortage occurred (Scheil et al., 2011).

At a molecular level, Hsps thus play a crucial role in the ability of terrestrial snails to survive the challenges of heat and desiccation in their environment. In this context, one should expect an extensive and rapid Hsp response in those species that inhabit extreme environments in order to help to preserve essential proteins, as well as to reduce the number of damaged proteins and associated energetic costs and, therefore, enhance the chance of survival under stressful conditions. In two *Sphincterochila* species living in arid habitats, Mizrahi, Heller, Goldenberg, and Arad (2011, 2012a) found an induction of Hsp70 and Hsp90 in foot and kidney tissue on exposure to elevated temperatures. In both species, however, the regulation of Hsps happened to be dependent on the reproductive status, which they regarded as indicative of a trade‐off between the synthesis of Hsp70 and Hsp90 and reproductive success. Therefore, on the one hand, a constitutively high Hsp level presumably should reduce the impairment caused by environmental threats. However, on the other hand, as production and a constantly high level of Hsps also imposes costs on the organism, evolution should favor an “emergency service” that leads to an induction of a considerable Hsp response whenever necessary, but does not keep Hsps at a high constitutive level (Köhler et al., 2009). Mizrahi et al. (2010) even found that evolution selects snails for reduced Hsp70 expression in harsh environments. Also, Mizrahi, Heller, Goldenberg, and Arad (2012b) found reduced expression of Hsps in the desert‐living *Sphincterochila zonata* and interpreted this as being indicative of a strategy that avoids the fitness consequences of continuous Hsp upregulation. Similar results have been published for other stress‐tolerant populations of numerous taxa in addition to snails (Arts et al., 2004; Bettencourt, Feder, & Cavicchi, 1999; Haap & Köhler, 2009; Köhler, Eckwert, Triebskorn, & Bengtsson, 1999; Köhler, Zanger, Eckwert, & Einfeldt, 2000; Krebs & Feder, 1997; Lansing, Justesen, & Loeschcke, 2000; Sørensen, Dahlgaard, & Loeschcke, 2001; Sørensen, Michalak, Justesen, & Loeschcke, 1999; Zatsepina et al., 2001). Despite of these studies which, in their entirety, suggest a general principle, Kotsakiozi et al. (2015) found the constitutive level of Hsp70 in six *Codringtonia* species to negatively correlate with altitude and mean summer precipitation at the sampling localities. They concluded that, in this case, the different species seem to have adapted to harsher environmental conditions by maintaining higher constitutive levels of Hsp70. Additionally, constitutive Hsp70 levels in the foot tissue of *Cornu aspersum* increase with decreasing latitude of the sampling site and, thus, increasing average temperature (Gaitán‐Espitia et al., 2013). The same applied to populations of *Theba pisana* in Israel among which individuals from warmer habitats were more thermotolerant and exhibited higher constitutive levels of Hsp70 in their foot tissue (Mizrahi et al., 2016).

Thus, apparently, there are indications for distinct Hsp strategies dependent on adaptations to different habitats—in closely related species and even in populations of the same species. This conclusion is supported, for example, by Arad et al. (2010) who tested whether adaption to different habitats affected the endogenous levels of Hsps in foot, hepatopancreas, and kidney of the two related snail species, *Sphincterochila cariosa* and *Sphincterochila zonata*, and showed that both species developed distinct strategies of Hsp expression that may have consequences for their ecology and distribution. Troschinski, Di Lellis, et al. (2014) tested ten populations of *Xeropicta derbentina* from southern France for their capacity to induce Hsp70 after artificial exposure to elevated temperatures. Even though all these populations derived from a spatially limited area 20 km around Carpentras, populations exhibited differing strategies to respond to 8‐hr heat stress, ranging from strong induction of Hsp70 to almost no change compared to the control level. All these strategies were associated with cellular integrity of the hepatopancreatic tissue. Thus, even within a species, apparent local ecological peculiarities seem to result in microevolutionary consequences regarding snails' molecular adaptations to heat. Interestingly, these interpopulation differences in the constitutive and maximal Hsp70 levels correlated with the variation in the shell coloration pattern in these *Xeropicta* populations (Di Lellis et al., 2014) which, in turn, may have consequences for the average thermal capacity of individuals from these populations (if intensely pigmented shells actually lead to higher body temperature). Furthermore, this observation strengthened the concept of Hsps as epigenetic capacitors of phenotypic variation (Di Lellis et al., 2014) as variation in coloration correlated inversely with the Hsp70 level in a number of *Xeropicta derbentina* populations from southern France: There was little color variation in populations exhibiting high Hsp70 levels and greater color variation in populations with low Hsp70 levels. The capacitoring concept has originally been suggested in nonmolluskan species by Rutherford and Lindquist (1998), Roberts and Feder (1999), Queitsch, Sangster, and Lindquist (2002) and Rutherford (2003), primarily for Hsp90 and traits other than pigmentation.

As stressful conditions associated with the lack of water had previously been found to induce Hsp expression in different organisms, Mizrahi et al. (2010) conducted a study to investigate the role of Hsps in the desert snails *Sphincterochila cariosa* and *Sphincterochila zonata* during desiccation. The results suggest that these snails use Hsps as a part of their survival strategy during desiccation stress. In their study, Mizrahi et al. (2010) included an Hsp70 isoform (Hsp72), Hsp90, and small Hsps (Hsp25, Hsp30) that were differently expressed across different tissues. *Sphincterochila cariosa* showed a significant upregulation of Hsp90 in foot and kidney during desiccation, whereas Hsp25 was only moderately induced. In *Sphincterochila zonata*, the Hsp90 levels in foot and kidney remained unchanged, but in contrast, the expression of Hsp25 was significantly enhanced. In all tissues examined, the Hsp72 level was significantly higher in *Sphincterochila cariosa*, the more desiccation‐sensitive species, than in *Sphincterochila zonata*. Since Hsp90 is only induced in the kidney of *S. cariosa* by desiccation, it is assumed that Hsp90 may participate in several specific processes during desiccation stress. The Mizrahi et al. (2010) study also points out the importance of small Hsps in processes accompanying desiccation. There was an upregulation of small Hsps in association with the lack of water, and the response of Hsp25 and Hsp30 was more pronounced in *S. zonata* than in *S. cariosa*. The authors hypothesized that the rising Hsp25 levels in the foot tissue during desiccation contribute to either a mechanism that regulates the stabilization of the cytoskeleton, chaperoning activities in the cytoplasm and nucleus, or both. Additional potential roles of small Hsps during desiccation may be protection of kidney tissue from deleterious effects of high urea concentrations and high body osmolarity accompanying longer periods without external water supply (Arad, 2001). This is also supported by the finding that snails that are adapted to desert‐like habitats developed a distinct Hsp expression strategy in the kidney to improve their water economy, while they maintain lower standing stocks of small Hsps and Hsp70 in other tissues (Arad et al., 2010). Although the exact functions of small Hsps during desiccation are not completely understood, it is at least known that organisms use small Hsps to adapt to extreme environmental conditions (Mizrahi et al., 2010).

In a number of *Theba pisana* populations, drought resistance was higher in populations from hotter habitats (Mizrahi et al., 2015). Also, higher desiccation resistance correlated with higher constitutive Hsp74 in the foot. During desiccation, starting from the constitutive levels, Hsps were generally upregulated during desiccation but with a delayed and lesser response in the most desiccation‐resistant population (Mizrahi et al., 2015). Thus, it can be concluded that there is considerable involvement of the Hsp machinery, both in survival of experimental desiccation and as a part of the natural annual cycle of activity and estivation in desert snails (Arad et al., 2010). As well, as reported for the Hsp responses to elevated temperature above, there are indications that the most drought‐resistant species (Mizrahi et al., 2010) and populations (Mizrahi et al., 2015) refrain from an extreme upregulation of *hsp* expression.

### 5.2 The role of Hsps in the biochemistry of snail estivation

Estivation is important in many snail species enabling individuals to survive long periods of extreme temperatures and desiccation, and Hsps are considered to be important components of the estivation mechanism, particularly with respect to the transition from activity to estivation (Mizrahi et al., 2010), as well as regarding their survival strategy during and after arousal. In this context, it seems that shifting from estivation to activity might be even more stressful than estivation itself (Arad et al., 2010). As a rapid increase in Hsp70 and Hsp90 synthesis occurs in *Sphincterochila cariosa* during early stages of desiccation, it may be assumed that these proteins not only play an important role during the desiccation process itself but might also be essential for the entire estivation process (Mizrahi et al., 2010). In *Cantareus apertus*, however, no significant change in Hsp70 was found when estivating individuals were compared to control snails (Reuner et al., 2008).

The crucial point during estivation in snails is to avoid water loss from the soft body. A typical physiological adaptation is an increased body fluid osmolality achieved by the production of high concentrations of solutes (Storey & Storey, 2011). More than 30 metabolites, including phospholipids and amino acids, are elevated in the hemolymph of estivating *Theba pisana* (Bose et al., 2016). In snails, urea is usually the prevailing osmolyte, helping snails to retain body water during estivation (Arad, 2001). After arousal from estivation, the high urea concentration in the tissues of the snail's soft body facilitates water uptake because of the considerably elevated concentration difference. In this process of ionic and osmotic regulation, the kidney plays an essential role (Arad et al., 2010). Kidney cells respond to hyperosmolality by inducing the synthesis of different Hsps (Beck, Neuhofer, & Müller, 2000). In particular, Hsp70 seems to be associated with all adaptations to chronic hyperosmolality (Santos, Pullman, Chevaile, Welch, & Gullans, 2003). Arad et al. (2010) addressed the possible functions of two Hsp families in a more detailed way, suggesting that Hsp70 probably acts as molecular chaperone when cellular proteins are structurally damaged, whereas Hsp90 may be part of signal transduction. They also found differences in the endogenous Hsp levels of *Sphincterochila cariosa* and *Sphincterochila zonata* in a tissue‐dependent manner. This may indicate distinct Hsp expression strategies that might reflect the relative sensitivity of different tissues and their physiological function during estivation. In *Sphincterochila zonata*, which is more heat‐tolerant and had a significantly higher osmolality than *Sphincterochila cariosa*, kidney cells synthesized Hsps, thereby enhancing osmotic tolerance and allowing maintenance of hyperosmotic conditions, and hence with positive consequences for water retention during estivation and water uptake after arousal. Due to the remarkable differences in Hsp expression between active and estivating land snails, it can be concluded that activity and estivation appear to be two distinct physiological states with corresponding Hsp strategies (Arad et al., 2010).

Upregulation of Hsps may enhance survival under stress by protecting and rescuing essential proteins. Therefore, on the one hand, it can reduce energetic costs associated with protein damage and subsequent degradation. However, on the other hand, it may be deleterious for growth and development because of trade‐offs in energy allocation (Mizrahi et al., 2010). In the light of these potential restrictions, there is evidence that juveniles might respond to heat stress more efficiently than adult snails because juveniles have to keep their Hsp system actuated due to the necessity to chaperone nascent proteins during growth, and because of the energy demands of reproduction in mature individuals (Dittbrenner et al., 2009). Fitness consequences associated with the maintenance of high standing stocks of Hsps could play a key role in limiting a species' distribution. Individuals of species that regularly experience severe environmental stress might be subjected to selection for reduced Hsp expression (Mizrahi et al., 2010) and, in turn, have to adopt alternative mechanisms to cope with harsh environmental conditions in such a way that survival and reproduction is ensured (Arad et al., 2010).

### 5.3 Molecular responses to oxidative stress

As estivation is characterized by a hypometabolic state, transition from estivation to activity goes along with upregulation of the metabolic rate and an increase in oxygen consumption which can result in oxidative stress in arousing snails. The same applies to active snails that increase their respiration with increasing temperature. Oxidative stress can be generated by emerging oxyradical production, and those oxyradicals may attack lipids and proteins aggressively. To avoid cellular damage, proteins have to be protected from denaturation, which is accomplished by the Hsp machinery (Arad et al., 2010). Additionally, Arad et al. (2010) suggested that the induction of Hsp70 and of small Hsps in *Sphincterochila* snails in transition from estivation to activity was probably caused by the oxidative stress associated with the upregulated metabolic rate. In *Otala lactea*, antioxidant defenses are already activated during estivation, which allows the snails to deal with the oxidative stress that takes place during arousal when oxygen consumption and the mitochondrial production of reactive oxygen species rise rapidly (Hermes‐Lima, Storey, & Storey, 1998; Storey, 2002). In *Helix pomatia*, glutathione reductase activity and lipid peroxidation remain constant during estivation and the glutathione level even increased in estivating individuals, although estivation resulted in downregulation of catalase and glutathione peroxidase (Nowakowska, Świderska‐Kołacz, Rogalska, & Caputa, 2009). In *Xeropicta derbentina* that were exposed to different temperatures for 8 hr, Troschinski, Dieterich, Krais, Triebskorn, and Köhler (2014) and Dieterich et al. (2015) recorded the highest lipid peroxidation level at 38°C followed by a second peak at 45–48°C which they proposed to reflect a two‐phase antioxidant defense system. Troschinski, Dieterich, et al. (2014) found a high constitutive catalase level supplemented by a particularly high glutathione peroxidase level at 40°C which explained the rather low lipid peroxidation rate at around this temperature. The catalase level peaked at 43–45°C but decreased at higher temperatures, allowing high lipid peroxidase levels to occur at temperatures of 45°C and higher. Hence, snails seem to possess an elaborate system of antioxidant mechanisms that contributes to heat tolerance during both active and dormant phases.

## 6 CONCLUSIONS

**Figure 8:**
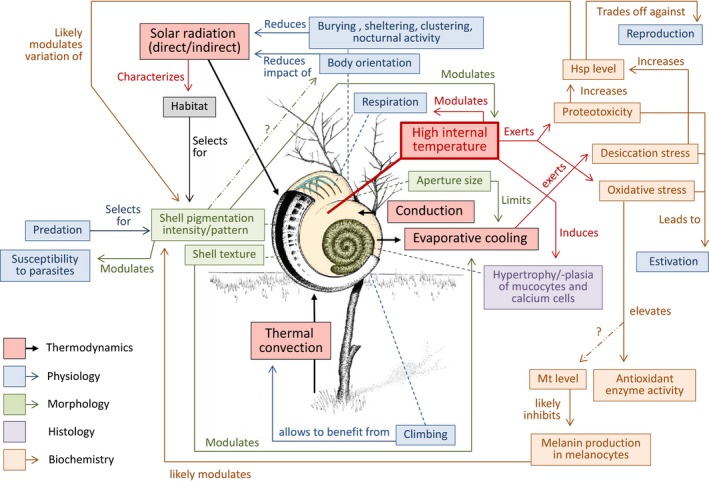
Integrative visualization of the networking interdependences of thermodynamics, snail biochemistry, physiology, histology, and the morphology of the shell, including back couplings as known so far. Thick solid arrows indicate energy transfer; dot‐dashed arrows are uncertain. Detailed explanation in the text

A suit of numerous mechanisms allows terrestrial snails to persist in hot and dry habitats (Figure 8). Aspects of thermodynamics, and behavioral, morphological, cellular, and molecular adaptations are interconnected and work together in a highly sophisticated system. The snails' survival usually does not depend on a single adaptation, but is based on a variety of modifications that have arisen during the evolutionary process. Differences even between closely related species of land snails imply that the evolved strategies are tailored precisely for each species and, sometimes, population, depending on the conditions they have encountered over time, and still do. Although broad knowledge on this subject has already been gained, future work calls for a completion of the entire picture and, thus, for a variety of experiments to be conducted. Such experiments necessarily need to refrain from setups that exclusively address monocausal explanations to account for the complexity of relationships as elaborated in this review. Statistical methods that are able to assist in detecting the relevance and the manner of combined action of factors related to snails' resistance to heat and drought are available today. It needs to be emphasized that land snails in hot and dry environments are among the best‐studied models in physiological evolutionary ecology. Nevertheless, both high‐end techniques and big datasets are required to answer the open questions that address these snails as thermodynamically controlled objects and ecologically determined biological entities that are subject to evolution in a rapidly changing world.

## CONFLICT OF INTEREST

None declared.

## AUTHORS' CONTRIBUTIONS

MS and H‐RK reviewed the literature and wrote the manuscript. RT contributed with discussions and manuscript editing. H‐RK prepared the figures and the final version of the manuscript.

## Data Availability

All data that are subject to this review paper have been published elsewhere. References including doi codes, as available, are given accordingly.
